# Evaluating the Feasibility of Using Brick Powder and Clay Powder in Cement Replacement

**DOI:** 10.3390/ma15228127

**Published:** 2022-11-16

**Authors:** Patryk Rumiński, Maciej Szeląg, Paulo de Matos

**Affiliations:** 1 Faculty of Civil Engineering and Architecture, Lublin University of Technology, 40 Nadbystrzycka Street, 20-618 Lublin, Poland; 2 Coordenadoria Acadêmica, Federal University of Santa Maria (UFSM), Cachoeira do Sul 96503-205, Brazil

**Keywords:** cement paste, brick powder, clay powder, support vector machine (SVM), physico-mechanical properties

## Abstract

The cement industry generates very large amounts of CO_2_ into the atmosphere. In recent years, there has been a search for alternative cementitious materials and micro-fillers that could partially or fully replace cement in cement composites without compromising their durability. This paper investigates the possibility of using brick powder (BP) and clay powder (CP) as a partial replacement for cement (up to 20% by weight) in cement paste. The raw materials were characterized, and the physical and mechanical properties of the modified cement pastes were studied, as well as their resistance to a short-term thermal shock at 250 °C. The study was supplemented by intelligent modelling of compressive strength using the support vector machine (SVM) algorithms. The results indicated a significant increase in tensile strength (up to 100%) and an increase in thermal resistance of cement pastes modified with BP and CP. The proposed SVM model had high accuracy (R^2^ = 0.90), indicating its suitability to predict the compressive strength of the modified cement matrix. This study complements the knowledge in the field of inter alia, the effect of a short-term thermal shock at elevated temperature on the properties of BP and CP modified cement paste, and the effect of BP, which, due to its grain size, plays more the role of a microfiller than a pozzolanic additive.

## 1. Introduction

With the beginning of the 21st century, global demand for building materials and cement production has grown rapidly, making it one of the major sources of increasing carbon dioxide emissions [1,2,3,4,5]. Current global concrete production is responsible for nearly 7.8% of nitrogen oxide emissions and 4.8% of sulfur oxide emissions [6]. In the past five decades, production activity has increased dramatically from 594 million tons of cement in 1970 to 2284 million tons in 2005, with the vast majority of production taking place in Asian countries [7,8]. It is estimated that by 2050, annual global cement production will be as high as 4380 million tons [9]. The production of excessive CO_2_ into the atmosphere leads to many negative climate variables on the economy and building structures. The process of degradation is one of the factors shortening the lifespan of concrete structures [10,11].

Given the current data, an important goal is to develop methods to reduce greenhouse gas emissions in concrete production, i.e., new kiln technologies, geopolymer cement [9], assessing the impact of mix design age on reducing greenhouse gas emissions [12], replacing clinker with mineral additions in cement [13], and using waste or demolition products as a substitute for cement mass. These materials can be divided based on their origin into three groups: the construction waste generated during the facilities’ construction, waste from renovation, demolition, destruction caused by natural disasters and armed conflicts, and waste from the processing industry [14,15,16,17].

One potential recycled material to replace cement in concrete production is brick powder (BP). Using BP as a substitute for cement has good economic benefits, reduces carbon emissions, and improves the material strength parameters [18,19,20,21,22,23]. Ma et al. [24] performed a study using waste brick powder (WBP) as additional cementitious material. They showed an improvement in the degree of hydration and refinement of the pore structure in the cement matrix in the case of high WBP fineness, where the pozzolanic activity of the WBP also increased with increasing fineness. It was verified that the compressive strength at a higher WBP fineness than cement fineness increased with an increase in WBP content up to 15%, and the maximum activity index was about 90%. At WBP fineness close to or similar to that of cement, the compressive strength decreases as the fineness ratio increases. Flexural strength decreases linearly with increasing WBP content in the mix.

Xue et al. [25] experimentally investigated the effect of content and fineness of BP on the strength of cement mortar. The study showed that the addition of BP reduced the early compressive strength more than the early flexural strength. It was assumed that the optimal content of BP in mortar could be 10–20%. Mortar strength can be improved by increasing the content of BP particles smaller than 20 μm. Similar strength results have been obtained by other researchers who made mortars with other percentages of BP in the mix [26,27,28,29].

A similar waste material is clay powder (CP), which, in combination with cement, creates the so-called alumina cement (AC). This product is made by grinding clay clinker and then sintering the limestone with bauxite without gypsum [30,31,32]. A small amount of AC makes the microstructure more compact, rapidly increases the compressive strength and adhesion to the concrete aggregates, and makes setting time shorter [33,34,35,36,37].

Zieliński and Kierzak [33] performed tests to determine the effect of cement with the AC addition on the basic physical and mechanical properties of cement mortars. The CEM I 42.5R cement and three types of AC with the following content of 40%, 50%, and 70% of Al_2_O_3_ were used for the tests. Additionally, mixtures containing various types of AC were made with percentages of 4%, 6%, 8%, 10% and 12%. Scientists showed that the initial and final binding process accelerates with the increasing amount of Al_2_O_3_ in the mix with CEM I 42.5R. It was found that the 6% content of AC gave a similar effect of immediate sample binding and better grain adhesion compared to 12% low AC. In summary, AC producers can reduce production costs and market prices in the case of replacing low-alumina cement with high-alumina cement.

Castillo et al. [38] examined the effect of an additive in the form of a clay powder as a cement replacement in concrete production. They paid special attention to reinforced concrete elements that are exposed to steel corrosion and are located in a marine area. In the study area, there are buildings that were designed without durability criteria, in which the use of pozzolanic materials is being considered. For this purpose, a reference concrete without the additive and concrete with a cement substitute in the form of a clay powder with a percentage of 5%, 15%, 25%, and 30%, respectively, were compared. It was found that as the CP additive increases, the fluidity of the mixture decreases by absorbing water in the mixture, making the mixture less workable. An increase in compressive strength by 25% for samples with CP addition was also obtained. A reduction in the pore volume of the cement matrix in CP-added specimens was observed by filling pores with fine CP particles.

Rani and Jenifer [39] have studied the behavior of CBP in a concrete mix. The crushed grounded clay was a product from the demolition of masonry buildings. It was grounded in the laboratory and added to cement-based mixtures as a replacement. Concrete recipes with 10%, 20% and 30% CBP replacement were made and tested for mechanical properties. It was found that compressive, flexural, and tensile strengths increased at 10% and 20% CBP content in the mix, but for 30% there was a decrease to a value comparable to the reference concrete. With this procedure, concrete becomes environmentally friendly as the amount of construction waste, i.e., clay powder, in the environment is reduced through its use. It was noted that the w/c ratio is kept constant with an increase in the percentage of CBP.

A study of the replacement ability of parched and grounded clay powder in cement matrix was also conducted by Zhu et al. [40]. A series of specimens with varying percentages of CP and silica fume were made. It was found that flexural strength at day 3 and 28 decreased by 27% and 18%, respectively, when the CP percentage ratio increased from 9% to 27%. This is due to the fact that the cement has a higher pozzolanic activity than CP. The decrease in compressive strength was small. As the amount of CP increases, the resistance of the concrete to the negative effects of chloride ions decreases. For some concrete with 10% CP replacement, the chloride ion charge transferred to the mix increased by 109% and for 25% CP replacement it was 179%.

The prediction of mechanical properties of cementitious composites, especially compressive strength, is of great practical importance. The ability to predict the behavior of a cementitious material under given operating conditions on the basis of information on its composition brings a number of tangible benefits, such as lower costs of material testing or assessment of durability and wear [41]. The standard predictive approach is based on simple correlation relationships, but this approach is inefficient and can often result in large prediction errors [42]. In recent years, there has been a very strong development of sophisticated methods of data analysis based on machine learning algorithms. Their main advantage is that the algorithm learns and increases its prediction accuracy as new data are supplied to the model, which allows accurate prediction of even nonlinear problems [43]. For cementitious materials, the support vector machines (SVM) approach is particularly popular for this purpose. For example, in [44], SVM was used to predict the mechanical performance of the cement paste based on the cracking patterns properties. The prediction accuracy of the analyzed models was in the range of 91–99%. In [45], the exposed temperature of fire-damaged concrete structures was predicted by means of the SVM. Depending on the cases studied, an accuracy of 53–89% was obtained.

In this study, the influence of BP and CP on the physical and mechanical properties of cement paste was investigated. Three types of samples were tested: standard samples made of pure cement paste, samples with the BP replacing a part of the cement mass in the amount of 5%, 10%, 15% and 20%, and samples with the CP replacing a part of the cement mass in the same amounts as in the BP samples. In the first stage of the research, the raw materials were characterized, and then a number of physical and mechanical properties of the modified cement pastes were examined. Moreover, the resistance of the tested materials to a short-term thermal shock at a temperature of 250 °C was investigated. The predictive modeling of compressive strength was also performed, based on the knowledge of BP and CP content, and information om whether the cement matrix was subjected to thermal loading or not. For this purpose, the SVM regression approach was used. The test results confirmed the possibility of using BP and CP in the production technology of cement composites.

The conclusions and analyses presented in the paper are mainly limited to the tested material and test conditions, i.e., BP and CP with specific properties, especially in terms of the particle size distribution and chemical composition; the content of BP and CP in the range of 0–20% used as a mass replacement for cement; water to cement ratio (*w*/*c*) in the range 0.4–0.5; load temperature in the range of 20–250 °C; and the size of the test specimens equal to 40 × 40 × 160 mm.

A scientific novelty in the paper, compared to other works on similar topics, is the evaluation of the properties of BP- and CP-modified cement pastes subjected to a short-term thermal load at an elevated temperature. Another advantage is the use of the SVM regression approach to predict the compressive strength of cement pastes with BP and CP. In addition, the vast majority of works available in the literature use BP with a finer grain size than in the studies conducted, which puts BP in the role of a non-reactive micro-filler rather than a pozzolanic additive.

## 2. Materials and Methods

### 2.1. Materials

Of the commercially available hydraulic binders, the CEM I 42.5 R Ordinary Portland Cement (OPC) was used in the study. The main ingredients are Portland clinker (95% content) and sulfate raw material, which act as a setting time regulator with a 5% content. The specific surface area (s) was tested in Blaine’s apparatus, and the value obtained was s = 4139 cm^2^/g. One of the materials serving as a replacement for cement was brick powder (BP), obtained from the manufacturer in the form of a fine red powder, which (s) tested in Blaine’s apparatus is s = 3337 cm^2^/g. The second material was clay powder (CP). The material was used in the form of dried and ground at the production stage, loose clay of brown color, with a fraction of 0–1 mm. The specific surface area measured in Blaine’s apparatus was equal to s = 4135 cm^2^/g.

Nine recipes of cement mixtures based on the cement alone and mixtures with a different BP and CP content were prepared for the study. The modification consisted of replacing the cement mass with BP or CP in the amount of 5%, 10%, 15%, and 20%, respectively. The series were marked as P0 (reference series—a pure cement paste); CP5, CP10, CP15, and CP20 series with CP; BP5, BP10, BP15, and BP20 series with BP. The number in the designation indicates the percentage replacement of cement by the BP or CP.

The mixing process was the same for all nine recipes. The paste was prepared by pre-mixing the bulk ingredients, then water was added. The *w*/*c* (water/cement) ratio in the series of pastes using CP was 0.4; for the BP replacement samples it was 0.5. The prepared paste was filled into tripartite molds in two layers, and compacted on a vibrating table. Nine sets of rectangular specimens of 40 × 40 × 160 mm were made in the molds in accordance with the PN-EN 196-1:2016-07 [46]. After 24 h, the samples were unmolded and then placed in a water bath, where they were conditioned for another 28 days.

After the maturation period, the samples were taken out of the water bath, and some of them were subjected to a short-term thermal shock (TS) by putting them into an oven pre-heated to 250 °C for a period of 4 h. In this case, the aim was to study the performance of the modified cement pastes under short-term thermal shock in a temperature range in which the cement matrix is still relatively chemically stable. The main changes that occur in the cementitious material at temperatures up to 250 °C are mainly the evaporation of free water and large deformations related to swelling in the heating phase and shrinkage in the cooling phase, which results in the formation of cracks and micro-cracks.

### 2.2. Methods

**Figure 1:**
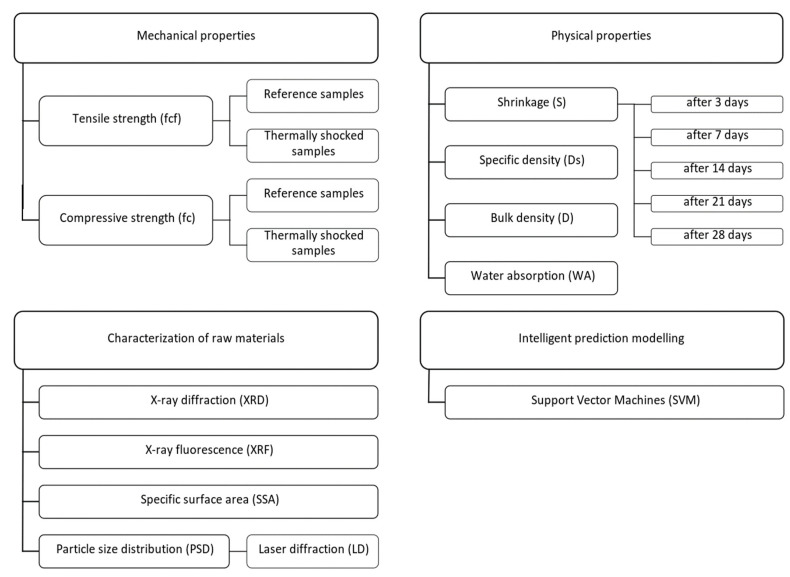
Flowchart of the test methods.

The methods used in the study are listed below and shown schematically in Figure 1.

Characterization of raw materials:

- X-ray diffraction (XRD),
- X-ray fluorescence (XRF),
- particle size distribution (PSD) by the laser diffraction (LD) method,
- specific surface area (SSA) according to Blaine’s method.

Testing of mechanical properties:

- tensile strength (f_cf_) in the bending test,
- compressive strength (f_c_),
- tensile strength after exposure to the elevated temperature (f_cfT_),
- compressive strength after exposure to the elevated temperature (f_cT_).

Testing of physical properties:

- shrinkage (S),
- specific density (D_s_),
- bulk density (D),
- water absorption (WA).

Intelligent prediction modelling:

- Support vector machines (SVM).

The PSD test of CEM, BP, and CP was carried out using the laser diffraction (LD) method. It was determined using a Mastersizer 2000 equipment (Panalytical, Malvern, UK). The test was carried out using isopropanol as a dispersing agent.

XRD was used to determine the phase composition of the materials studied. The analysis was performed with an X’pert PRO diffractometer (Panalytical, Almelo, The Netherlands) with a PW 3050/60 goniometer. The tests were carried out on samples grounded in an agate mortar. The analysis was carried out with CuKα radiation over an angular range of 5–65° 2θ, with a measurement step size of 0.03° 2θ and a time of 4 s per step.

The chemical composition of CEM, BP, and CP was determined using the X-ray fluorescence (XRF) method. Prior to testing, samples were dried to a constant weight and then grounded in an agate mortar. The powder was transferred to plastic containers for analysis. The test was carried out using an Epsilon 3 spectrometer (Panalytical, Alamo, The Netherlands).

The SSA of CEM, BP, and CP was determined using Blaine’s apparatus in accordance with [47].

The tensile strength (f_cf_) test was carried out on the reference samples and samples after the thermal shock and cooled to room temperature (~20 °C). The test was carried out after 28 days using an MTS 810 strength machine (MTS Systems, Eden Prairie, MI, USA) with a 10 kN head in a three-point bending scheme. The samples were placed in the strength machine, in which the position of the concreting surface was perpendicular to the direction of loading. After the test was completed, the halves of the broken samples were subjected to a compressive strength test. The tensile strength was calculated according to the formula in the standard [48]. The results are the arithmetic mean of three specimens for each series. 

The compressive strength (f_c_) test was conducted in accordance with the EN 12390-3 [49]. The test was performed using a Advantest 9 strength machine (Controls, Milan, Italy) with a maximum pressure of up to 250 kN, with overlays used for compressive strength testing on 40 × 40 × 40 mm specimens. The compressive strength was calculated according to the formula in the standard [49]. The results are the arithmetic mean of six specimens.

Thermal loading of the specimens in a furnace pre-heated to 250 °C caused damage to the structure, mainly by cracks due to the volumetric deformations that occurred. During the thermal loading process, the swelling of the specimens occurred, while during the cooling phase the specimens were subjected to excessive shrinkage. As a result of the heating process, the energy created is a source of change of free water molecules into water vapor, which, under the influence of increased pressure in the pores and capillaries, leads to the expansion of cracks in the paste structure. Thermal loading was aimed at determining the effect of a thermal shock on the mechanical properties of modified cement pastes.

The linear shrinkage (S) test was carried out with the Graf-Kaufman apparatus (EMEL, Warszawa, Poland). Samples were tested after 3, 7, 14, 21, and 28 days. The reference measurement was made immediately after the samples were unmolded, i.e., 24 h after forming. The samples for this study were conditioned under air-dry conditions (RH ≈ 50%, temperature ≈ 20 °C). The results obtained are the arithmetic mean of three samples for each series.

Specific density (D_s_) was determined using the pycnometric method. Crushed paste samples were grounded in a ball mill. Pycnometers were filled with liquid alcohol with a density of δ_p_ = 0.835 g/cm^3^. The prepared pycnometers with samples were vented in a vacuum dryer. The test was carried out for BP, CP, and modified cement paste samples.

Apparent density (D) was determined after 28 days in accordance with the EN 1015-10 [50]. Samples were weighed on a laboratory scale, and dimensions were measured with a caliper. The results are the arithmetic mean of three samples for each series.

Water absorption (W_A_) was calculated by measuring the weight of the samples after 28 days in water conditions and after drying to a constant weight. Calculations were made for three samples for each series. Tightness (T) was calculated as the ratio of the apparent density (D) to specific density (D_s_). Total porosity (P_o_) was calculated using the percentage difference in tightness. The P_o_ and T results are the arithmetic mean of three samples for each series.

This paper also considers the problem of f_c_ prediction based on material data and other technological variables. For this purpose, the SVM regression approach was used. This approach is recommended for developing regression models for low-dimensional data sets [51], in comparison to other approaches, i.e., the artificial neural networks (ANN) or self-organizing maps (SOM). Inputs used the percentage content of BP and CP in the cement matrix, and whether the material was subjected to thermal loading or not. Output was the fc value. A number of SVM models were analyzed, selecting the most optimal one for further analysis, i.e., the one with the highest prediction accuracy, expressed by the root mean squared error (RMSE) value. The model parameters that were subjected to the optimization were: kernel function (linear, quadratic, cubic, Gaussian), kernel scale (0.001–1000), epsilon (0.006–600), box constraint (0.001–1000), and standardization data (true, false). The analysis was carried out in the Matlab software.

## 3. Results and Discussion

### 3.1. Cement, Brick Powder, Clay Powder—Basic Characteristics

#### 3.1.1. Physical, Chemical, and Phase Properties

The specific density of CEM was 2.93 g/cm^3^, while the specific surface area tested with Blaine’s apparatus was 4139 cm^2^/g. The specific density of BP was 2.67 g/cm^3^, and the specific surface area of BP was 3337 cm^2^/g. In contrast, the specific density of CP was 2.72 g/cm^3^, and the specific surface area of CP was 4135 cm^2^/g. The CEM and CP used have a very similar specific surface area, while BP has a lower specific surface area by 19.4%. The higher specific surface area of CEM and CP gives the material greater reactivity, resulting in a faster binding process in an early period. The lower specific surface area of BP indicates a lower particle size, which leads to improved properties of the mixtures, i.e., increased workability and reduced water demand.

**Figure 2:**
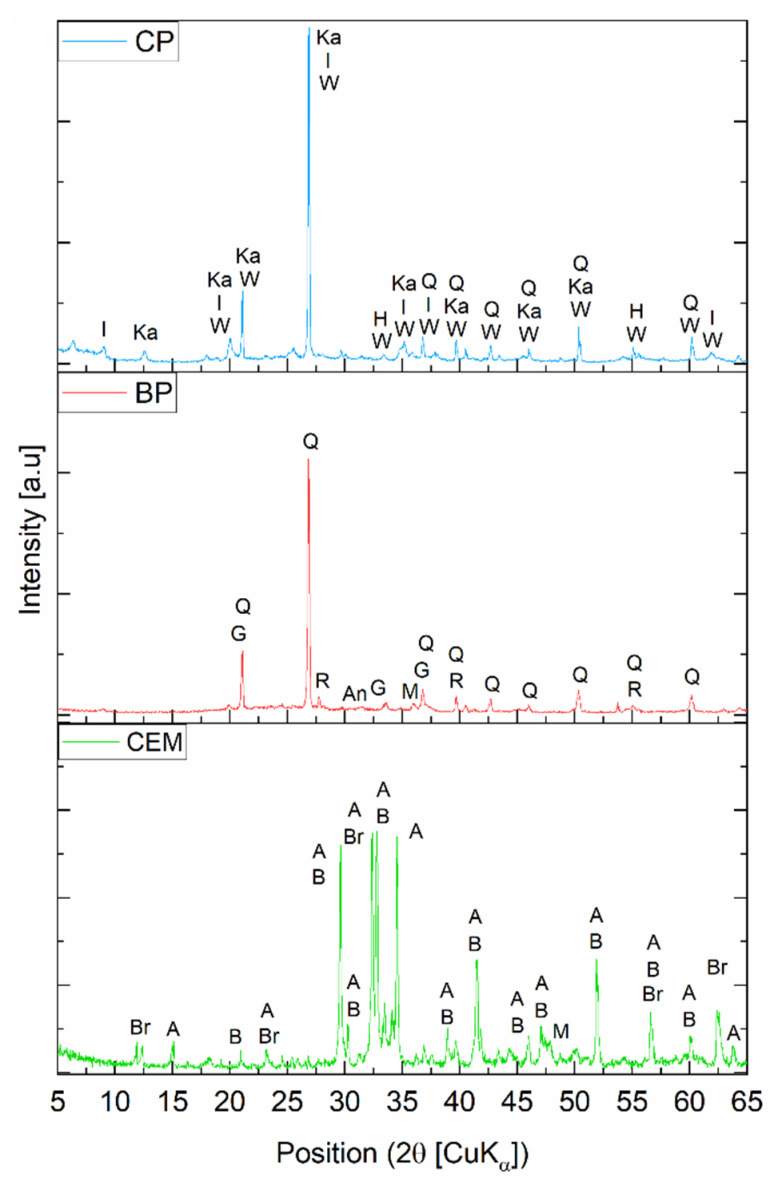
XRD pattern of the CEM, BP, and CP; A—alite, An—anhydrite, B—belite, Br—brownmillerite, G—grossular, H—hematite, I—illite, Ka—kaolinite, M—magnetite, R—rutile, Q—quartz, W—vermiculite.

The crystalline phases analysis on the XRD pattern for CEM, BP, and CP is shown in Figure 2. The chemical composition determined by the XRF method for CEM, BP, and CP is shown in Table 1.

The predominant elements in the composition of BP are SiO_2_, which accounts for 59.3%, aluminum oxide (Al_2_O_3_), which accounts for 14.1%, and iron oxide (Fe_2_O_3_) at 7.6%. The silica present in the BP may react with the hydration products of the cement to form a secondary C-S-H phase. Trace elements were also identified, e.g., SO_3_, TiO_2_, Cr_2_O_3_, MnO, NiO, ZnO, Rb_2_O, ZrO_2_, Ag_2_O, and BaO, in a total amount of 1.2%.

The main components of CP are SiO_2_ with a content of 56.1%, Al_2_O_3_ (14.7%), and Fe_2_O_3_ (8.6%). The high content of magnesium oxide may have a negative effect on the modified paste with CP and can lead to the formation of cracks. In the chemical composition of CP, trace amounts of elements were detected, which do not affect its physico-chemical properties. These include: TiO_2_, MnO, NiO, ZnO, Rb_2_O, BaO, ZrO_2_, Ag_2_O, with a total content of 1.2%.

#### 3.1.2. Particle Size Distribution

**Figure 3:**
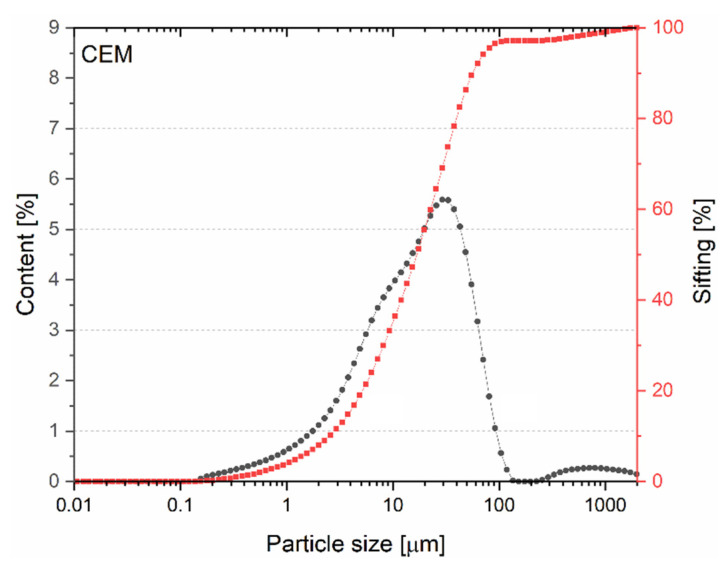
Particle size distribution of CEM.

The results of the CEM particle size distribution obtained using the LD method are shown in Figure 3. The material is dominated by a fraction with a size in the range of 1–100 μm, accounting for 93.3% of all grains. Overall, 50% of the grains of the cement used were found to be smaller than 17.9 μm (D_x50_ = 17.9 μm), and 10% of the grains were in the range of 0.01–2.69 μm. The content of large grains larger than 100 μm, accounting for 3.1%, may indicate the phenomenon of grain clumping.

**Figure 4:**
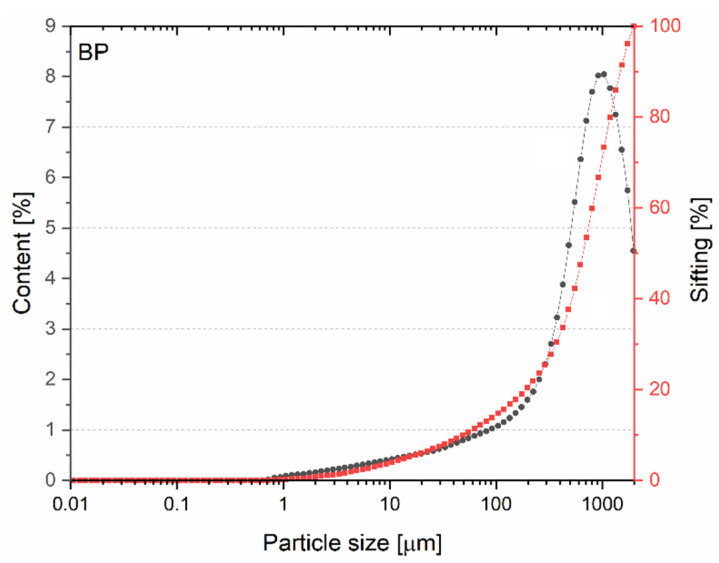
Particle size distribution of BP.

The BP particle size distribution curve (Figure 4) shows that grains with dimensions in the range of 0.01–52.8 μm account for 10% of the total content. Overall, 50% of BP grains were smaller than 709 μm (D_x50_ = 709 μm). The presented higher percentage of large BP fractions relative to the percentage of large cement fractions indicates that BP is likely to act as a micro-filler (micro-aggregate). Only the smallest grains will be characterized by relative reactivity with the cement matrix components.

**Figure 5:**
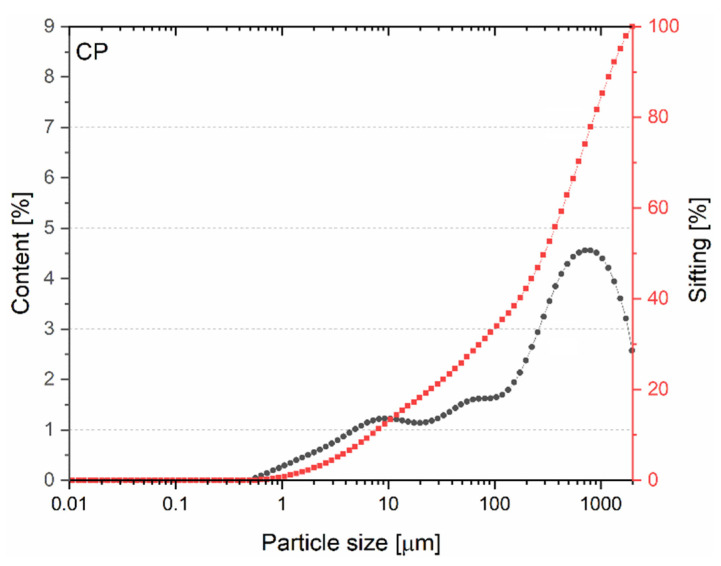
Particle size distribution of CP.

The particle size distribution figure (Figure 5) shows that the use of CP grains is similar in nature to that of BP grains. In the case of CP, grains smaller than 316 μm (D_x50_ = 316 μm) account for 50% of the volume share, while 10% of the grains occur in the 0.01–7.37 μm range. As with BP, as a result of the significant content of grains larger than 100 μm, not much pozzolanic activity is expected.

### 3.2. Physico-Mechanical Properties of Hardened Cement Paste

#### 3.2.1. Physical Properties

The physical properties of cement pastes are shown in Table 2. The CP caused a decrease in the apparent density of cement pastes by 1.8% on average. The most significant decrease (of 3.8%) was recorded for the CP5 series. As the CP content increased, the value of specific density decreased until the CP20 series, for which practically the same D was obtained as for the reference P0 series. The increase in CP content resulted in an increase in total porosity. The most significant increase was observed for the CP20 series (36%). The increase in total porosity is directly related to the decrease in the tightness of the samples. A decreasing trend was shown for the CP5, CP10, and CP20 series. A 1% increase in tightness was recorded for the CP15 series, a value that can be considered not quite correct given the increase in density in this series. Given the decreasing value of tightness with increasing CP content, it is inferred that CP causes an increase in cement paste porosity. This is due to the higher content of the smallest particles fractions in the cement (Figure 3). The CP also caused an increase in the water absorption of the cement paste in each series of samples. For CP20, an increase of 21.7% relative to P0 was recorded.

When BP was used, the BP5, BP10, BP15, and BP20 series had a lower apparent density compared to P0, with the largest recorded decrease in D_s_ equal to 4.8% for BP5. The average decrease in D_s_ for BP samples is 3.8%. A decrease in specific density was obtained for BP5, BP10, and BP15, while BP20 showed an increase of 1.6%. A decrease in tightness was recorded in the BP5, BP15, and BP20 series, while in the BP10 series, the parameter did not change compared to the P0 reference series. An increase in the percentage of BP affects the increase in porosity, the highest value of which was recorded for the BP20 series (35.5%). This phenomenon has an adverse effect on the mechanical strength of the material, since it is well known that as the porosity of the cement composite increases, its compressive strength will decrease. Finally, similar water absorption was obtained in all series modified with BP (24.5–25.1%). The emerging disturbances in the trend of the obtained results may be due to imperfections at the stage of forming the samples and possible differences in the degree of compaction.

**Figure 6:**
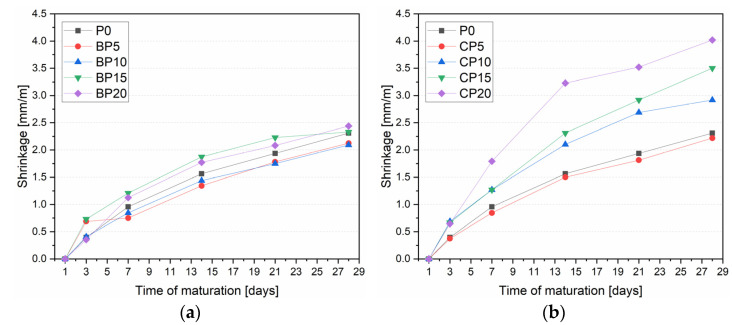
Linear shrinkage of BP (**a**) and CP (**b**) modified cement pastes.

Figure 6 shows the shrinkage of the samples at 1, 3, 7, 14, 21, and 28 days. The measurement taken on the first day was the reference measurement. After 3 days, the BP20 series showed the smallest shrinkage equal to 0.35 mm/m, 10.6% less than P0. Shrinkage increases of 73.5% and 84.0% were recorded for the BP5 and BP15 samples, respectively. After 7 days, the shrinkage of the BP15 and BP20 series was 26.1% and 17.4% higher than P0, respectively, and a similar relationship persisted in subsequent ages. Cement pastes with 5% and 10% of BP content from day 7 to the end of the period investigated obtained the lowest shrinkage values. At 28 days, the BP10 series had the lowest shrinkage value of all series, equal to 2.09 mm/m. In contrast, the BP20 series had the highest shrinkage value of 2.44 mm/m. The average linear shrinkage increment for all mixtures with BP was 8.2%. Considering the current values, the optimal percentage of BP in the mix is 10%; above this range, the kinetics of shrinkage increment increases, which is a negative phenomenon. This is confirmed by studies by other researchers [52], in which the increase in linear shrinkage increased with a higher percentage of BP content in the mix.

The series with CP showed that an increase in the content of CP in the mix negatively affects the linear shrinkage. After 7 days, the shrinkage value of the P0 reference sample was 0.95 mm/m, against which only the CP5 series achieved a lower value (by 11.9%). The CP10 and CP15 series were characterized by a higher shrinkage of 32.6%, while for the CP20 series, the shrinkage increase was 87.1% relative to the P0. Test results after 14, 21, and 28 days confirm the earlier trend. For the CP5 series, the results at each successive age were characterized by a smaller increase in shrinkage relative to the P0. For the CP10, CP15, and CP20 series, a significant increase in linear shrinkage values was shown. For the CP20 series, it showed the largest increase in linear shrinkage at 14 days, equal to 106.6% relative to P0. At 28 days, the CP20 series was shown to have the highest shrinkage value, equal to 4.0 mm/m. The average increase in linear shrinkage for all mixes with CP was 41.9%. The study shows that a 5% content of CP in the mix relatively reduces the shrinkage value throughout the investigated period. Hay et al. [53] observed similar shrinkage increase relationships for higher CP content in the mix.

The probable reason for the above relationship (for both BP and CP) is that a large part of the material used is unreactive with the hydration products of the cement and acts as a micro-filler, as also indicated by the grain size distribution curve of BP and CP. In this case, as the amount of material used increases, less and less water reacts with the binder. In the experiments conducted, the amount of water used in each series was the same, thus, a larger amount of water remained free at the initial stages of hydration, which intensified the drying process and drying shrinkage of the modified cement paste.

An increase in the content of CP and BP in cement pastes generally negatively affects the physical parameters of the samples. It should be noted that an increase in the content of CP in the mix clearly affects the increase in linear shrinkage. Taking into account a 5% content of CP, a reduction in early shrinkage relative to pure cement paste was demonstrated. In the case of specimens with BP, a much smaller upward trend was found relative to the CP samples. For the sake of clarity of Figure 6, the standard deviation for the BP and CP series is shown in Table 3. The occurrence of larger grains of CP and BP particles negatively affected the tightness of the mixtures, with an increase in the overall porosity of the samples relative to the P0. After the application of BP and CP, the water absorption of cement pastes also increased.

#### 3.2.2. Mechanical Properties

##### Compressive Strength

**Figure 7:**
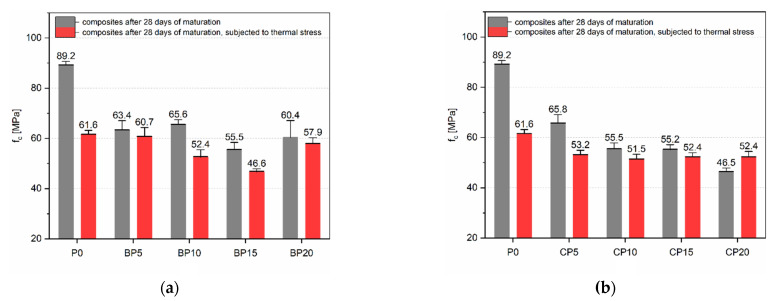
Compressive strength after 28 days: (**a**) BP series; (**b**) CP series.

The highest compressive strength (f_c_) for the BP series was obtained in the BP10 series (65.6 MPa) for samples not subjected to the thermal loading (Figure 7). The highest strength result with specimens subjected to the short thermal load (f_cT_) was obtained for the BP5 series with a value of 60.7 MPa, which represents 98.6% of the value of the reference cement paste (P0). The lowest f_c_ was recorded for the BP15 series equal to 55.5 MPa and f_cT_ of 46.6 MPa, which represents 75.6% of the strength of the reference series (P0). In the conducted tests, an increase in BP content in the cement paste mixtures resulted in a decrease in compressive strength. The BP20 series showed an increase in f_cT_ equal to 57.9 MPa, compared to the decreasing trend of the P5, P10, and P15 series. No increase in strength after the thermal shock was detected in the BP series.

The CP series (Figure 7) shows that the CP5 series has the highest f_c_, with a value of 65.8 MPa. The highest recorded f_cT_ is 53.2 MPa, which represents 86.4% of the f_cT_ value of the P0. It is worth noting that all the f_cT_ results of the mixtures with CP, differ slightly from each other, and the maximum difference between them is only 1.7 MPa. Comparing the CP series results to the BP series, it can be seen that the spread of results for BP is much larger. The lowest f_c_ value was obtained for P20 (46.5 MPa). In this case, the short-term thermal load caused a significant increase in f_cT_ to a value of 52.4 MPa. 

The results confirm previous predictions—that both BP and CP used in the tests play more the role of a microfiller than a reactive form of addition. It is worth noting that the largest decrease in f_c_ after the thermal loading occurred for the unmodified series (P0)—a 31% decrease in strength. For the cement pastes with BP and CP, the decrease in f_c_ after thermal shock was much smaller, averaging 11% and 6%, respectively. Ceramic materials inherently have a high resistance to elevated temperatures and their addition to cementitious composites generally improves their thermal resistance. This is due to the sheer thermal stability of the ceramic material, as well as the robust ITZ zone between the grains of the ceramic material and the cement matrix, as also confirmed by other studies [54,55].

##### Flexural Strength

**Figure 8:**
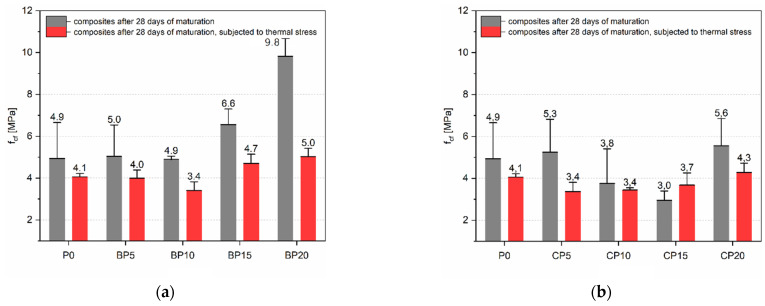
Tensile strength after 28 days: (**a**) BP series; (**b**) CP series.

A particularly favorable effect from the use of BP was obtained in the context of f_cf_ (Figure 8). The highest tensile strength (f_cf_) for the BP series was obtained for the BP20 series, which is 9.8 MPa, and this is an increase of 98.7% over the P0 series. The reference paste, BP5 and BP10 series, show f_cf_ at similar levels, i.e., 4.9–5.0 MPa. The BP10 series obtained the lowest tensile strength after the short-term thermal loading (3.4 MPa). There is an upward trend in f_cfT_ with the BP15 series (4.7 MPa) and BP20, for which the value is the highest among all samples (5.0 MPa). The average increase in f_cf_ for all series with the BP is equal to 33%, relative to the P0. In this case, the higher the BP content in the cement matrix, the generally higher the f_cf_ value. The reason for this can be attributed to two phenomena, i.e., first, the smaller amount of cement, in a fixed volume of material, with the increase in BP content promoting a reduction in the formation of microcracks during the testing period. Secondly, the BP grains present in the structure of the cement matrix can interlock against each other, increasing the strength.

The CP effect was already less favorable compared to the BP series. The highest f_cf_ was shown for the series with the highest CP content, and it was equal to 5.6 MPa (CP20). The lowest f_cf_ value was obtained for the CP15 series (3.0 MPa), which represents 59.7% of the f_cf_ value of the reference samples (P0). Due to the low apparent density (1.59 kg/m^3^) of the samples with 15% CP, their f_cf_ is the lowest of all the series (3.0 MPa). It is worth noting that the CP20 series has the highest f_cfT_ value of 4.3 MPa, which is 5.4% higher than P0. There was no leading effect of CP on f_cf_, as the two highest values were obtained for the lowest and highest CP content in the cement matrix (CP0 and CP20). In the case of f_cfT_, the presence of CP at low contents decreases the value of this parameter, after which, as CP content increases, f_cfT_ also increases. 

##### Brittleness

Table 4 shows the f_cf_ and f_c_ ratio of cement pastes with BP. The f_cf_/f_c_ ratio is also a basic measure of the brittleness of the material. Based on the following results, we checked what the BP effect on the brittleness of the hardened cement paste was. As the BP content increases, the brittleness of the material decreases, i.e., the f_cf_/f_c_ ratio increases. For non-thermally stressed specimens, the difference is as much as three times between the P0 and BP20 series. For thermally shocked samples, there is a similar relationship, but the difference between the extreme results is much smaller.

The CP also significantly affected the brittleness of the material for all the series (Table 5), but the differences between series are smaller than for BP. In comparison, the values of all CP series without thermal loading, are smaller compared to the BP series, indicating an increase in the brittleness of the cement matrix. For the specimens after thermal shock, a uniform decrease in the brittleness of the cement matrix with increasing CP content was noted. The correlations obtained indicate that due to the applied load, the BP and CP make the cement matrix fail less explosively.

### 3.3. Intelligent f_c_ Prediction Using SVM

**Figure 9:**
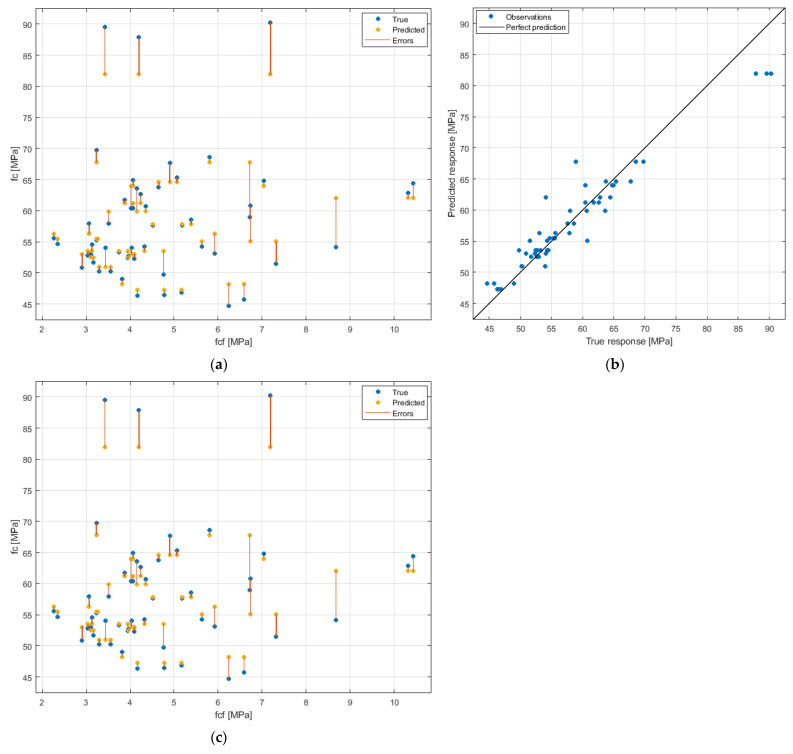
Prediction of compressive strength using the SVM: (**a**) f_c_-f_cf_ relation with prediction errors; (**b**) predicted-measured values relation; (**c**) distribution of residuals.

From a practical point of view, it is valuable to be able to predict f_c_ based on knowledge of the composition of the cement composite [56,57]. Considering the results obtained and the lack of a clear relationship, BP/CP content—f_c_, traditional linear or polynomial regression models—would have very low accuracy. Very good results with nonlinear problems are obtained using supervised machine learning algorithms. In this study, the SVM regression approach was applied to solve the f_c_ prediction problem. Details of the approach are described in the “Methods” section. The results are shown in Figure 9. The most optimal model was obtained for Gaussian function, kernel scale—0.5, box constraint—7.5612, epsilon—0.7561, and standardized data.

The RMSE value for the SVM model is 2.9813 MPa, the MSE (mean squared error) is 8.8883 MPa2, the MAE (mean absolute error) is 1.9980 MPa, and the R2 (coefficient of determination) is 0.90. The residuals distribution shows that the vast majority of them are below 4 MPa. Only in 6 cases out of 54 observations was the model distribution greater than this value. Considering the fact that the obtained f_c_ results are in the range of 45–90 MPa, the prediction of f_c_ using the SVM regression approach is characterized by a high accuracy.

## 4. Summary and Conclusions

The purpose of this study was to analyze the effect of cement mass replacement, in the form of BP and CP, on the basic physical and mechanical characteristics of the cement matrix. Due to the worrying topic of global warming and the continuous increase of cement production, there is an increasing demand for additions and admixtures for cement composites that will be environmentally friendly and become a good substitute for the common binder that is cement. The scientific novelties of the work include the study of the properties of cement pastes, with BP and CP subjected to a short-term thermal load and the use of BP with a larger grain size than in the vast majority of works available in the literature. On the basis of the conducted research, the following conclusions were formulated:

- The CP and BP reduced the compressive strength of the cement matrix. In the case of the thermal shock effect, a positive effect of BP and CP was observed, as f_cT_ was 11% and 6% lower than f_c_, respectively. For the P0 series, the difference was as much as 31%.
- As BP content increased, an increase in f_cf_ and f_cfT_ was observed. For the BP20 series, the value of f_cf_ was as much as twice that of P0. For CP-modified cement matrix, the effect on f_cf_ and f_cfT_ varied depending on the CP content.
- With an increase in BP and CP content, a decrease in the tightness and an increase in the overall porosity of the cement matrix were observed. This is the same as an increase in the water absorption of the modified samples. The highest water absorption was obtained for CP20 and was as much as 19% higher compared to P0.
- The CP series was characterized by higher shrinkage strain growth kinetics compared to the BP series. The BP10 series had the lowest shrinkage strain, reaching a shrinkage of 2.094 mm/m after 28 days. On the other hand, the content of BP and CP at 15% and 20% significantly increased the shrinkage strain compared to P0.
- An intelligent modelling approach was applied to predict f_c_ using SVM regression approach. The results show high accuracy of the model (R2 = 0.90), which can be used in practice to predict f_c_ of cement matrix with BP and CP.

Overall, it was found that the BP and CP have a positive effect on only some properties of cement pastes. A definite positive phenomenon is an increase in the tensile strength of specimens with the BP, as well as better properties in terms of resistance to short-term thermal shock.

## Figures and Tables

**Table 1:** Chemical composition of CEM, BP, and CP.

Content (%)			
Compound	CEM	BP	CP
SiO_2_	17.3	59.3	56.1
CaO	67.3	2.0	1.8
Al_2_O_3_	3.1	14.1	14.7
Fe_2_O_3_	3.5	7.6	8.6
K_2_O	-	3.4	3.9
MgO	-	2.3	2.9
SO_3_	4.0	-	-
Others	1.7	1.2	1.2
LOI *	3.1	10.1	10.8

* LOI—loss of ignition.

**Table 2:** Physical properties of cement pastes modified with CP and BP.

Series	P0	CP5	CP10	CP15	CP20	BP5	BP10	BP15	BP20
Specific density Ds [kg/m^3^]	2.44	2.38	2.39	2.32	2.44	2.41	2.32	2.39	2.48
Bulk density D [kg/m^3^]	1.66	1.60	1.56	1.59	1.55	1.62	1.59	1.58	1.60
General porosity P_o_ [%]	32.0	32.8	34.7	31.5	36.5	32.8	31.5	33.9	35.5
Tightness T [%]	68.0	67.2	65.3	68.5	63.5	67.2	68.5	66.1	64.5
Water absorption WA [%]	23.2	25.0	25.0	25.5	27.7	24.5	25.0	25.1	24.7

**Table 3:** Standard deviation of the linear shrinkage results.

Standard Deviation									
**Series**	**P0**	**BP5**	**BP10**	**BP15**	**BP20**	**CP5**	**CP10**	**CP15**	**CP20**
[%]	0.127	0.179	0.124	0.126	0.082	0.051	0.197	0.152	0.133

**Table 4:** Brittleness of cement matrix modified with BP.

Brittleness of BP				
Series	Reference Sample (R)	Samples Subjected to Thermal Stress (T)	Difference in Relation to P0	
	f_cf_/f_c_	f_cf_/f_c_	(R)	(T)
P0	0.055	0.066	-	-
BP5	0.080	0.066	44%	19%
BP10	0.074	0.065	34%	17%
BP15	0.118	0.101	114%	83%
BP20	0.162	0.087	193%	57%

**Table 5:** Brittleness of cement matrix modified with CP.

Brittleness of CP				
Series	Reference Sample (R)	Samples Subjected to Thermal Stress (T)	Difference in Relations to P0	
	f_cf_/f_c_	f_cf_/f_c_	(R)	(T)
P0	0.055	0.066	-	-
P5	0.080	0.063	44%	15%
P10	0.068	0.067	22%	21%
P15	0.053	0.070	−3%	27%
P20	0.119	0.082	116%	47%

## Data Availability

The data presented in this study are available on request from the corresponding author. The data are not publicly because they are part of a wider range of research that has yet to be fully published.

## References

[1] Andrew R.M. (2018). Global CO_2_ emissions from cement production. Earth Syst. Sci. Data.

[2] Barcelo L., Kline J., Walenta G., Gartner E. (2013). Cement and carbon emissions. Mater. Struct..

[3] Mehta P. Sustainable cements and concrete for the climate change era—A review. Proceedings of the Second International Conference on Sustainable Construction Materials and Technologies.

[4] Medina C., Banfill P.F.G., De Rojas M.S., Frías M. (2013). Rheological and calorimetric behaviour of cements blended with containing ceramic sanitary ware and construction/demolition waste. Constr. Build. Mater..

[5] Celik K., Meral C., Gursel A.P., Mehta P.K., Horvath A., Monteiro P.J. (2015). Mechanical properties, durability, and life-cycle assessment of self-consolidating concrete mixtures made with blended portland cements containing fly ash and limestone powder. Cem. Concr. Compos..

[6] Miller S.A., Moore F.C. (2020). Climate and health damages from global concrete production. Nat. Clim. Change.

[7] Taylor M., Tam C., Gielen D. (2006). Energy Efficiency and CO_2_ Emissions from the Global Cement Industry. Korea.

[8] Xiao J., Li W., Fan Y., Huang X. (2012). An overview of study on recycled aggregate concrete in China (1996–2011). Constr. Build. Mater..

[9] Hasanbeigi A., Price L., Lin E. (2012). Emerging energy-efficiency and CO_2_ emission-reduction technologies for cement and concrete production: A technical review. Renew. Sustain. Energy Rev..

[10] Medeiros R.A. (2018). Impact of climate change on the service life of concrete structures. Eco-Efficient Repair and Rehabilitation of Concrete Infrastructures.

[11] Stewart M.G., Wang X., Nguyen M.N. (2011). Climate change impact and risks of concrete infrastructure deterioration. Eng. Struct..

[12] Miller S.A., Horvath A., Monteiro P.J. (2016). Readily implementable techniques can cut annual CO_2_ emissions from the production of concrete by over 20%. Environ. Res. Lett..

[13] Habert G., Roussel N. (2009). Study of two concrete mix-design strategies to reach carbon mitigation objectives. Cem. Concr. Compos..

[14] Robayo-Salazar R.A., Rivera J.F., de Gutiérrez R.M. (2017). Alkali-activated building materials made with recycled construction and demolition wastes. Constr. Build. Mater..

[15] Wu Z., Ann T.W., Shen L., Liu G. (2014). Quantifying construction and demolition waste: An analytical review. Waste Manag..

[16] Malešev M., Radonjanin V., Marinković S. (2010). Recycled Concrete as Aggregate for Structural Concrete Production. Sustainability.

[17] Rasheeduzzafar K.A. (1984). Recycled Concrete-A Source for New Aggregate. Cem. Concr. Aggreg..

[18] Liu Q., Li B., Xiao J., Singh A. (2020). Utilization potential of aerated concrete block powder and clay brick powder from C&D waste. Constr. Build. Mater..

[19] Letelier V., Ortega J.M., Muñoz P., Tarela E., Moriconi G. (2018). Influence of Waste Brick Powder in the Mechanical Properties of Recycled Aggregate Concrete. Sustainability.

[20] Ge Z., Gao Z., Sun R., Zheng L. (2012). Mix design of concrete with recycled clay-brick-powder using the orthogonal design method. Constr. Build. Mater..

[21] Arif R., Khitab A., Kırgız M.S., Khan R.B.N., Tayyab S., Khan R.A., Anwar W., Arshad M.T. (2021). Experimental analysis on partial replacement of cement with brick powder in concrete. Case Stud. Constr. Mater..

[22] Naceri A., Hamina M.C. (2009). Use of waste brick as a partial replacement of cement in mortar. Waste Manag..

[23] Lin K.L., Wu H.H., Shie J.L., Hwang C.L., Cheng A. (2010). Recycling waste brick from construction and demolition of buildings as pozzolanic materials. Waste Manag. Res..

[24] Ma Z., Tang Q., Wu H., Xu J., Liang C. (2020). Mechanical properties and water absorption of cement composites with various fineness and contents of waste brick powder from C&D waste. Cem. Concr. Compos..

[25] Xue C., Qiao H., Cao H., Feng Q., Li Q. (2021). Analysis on the Strength of Cement Mortar Mixed with Construction Waste Brick Powder. Adv. Civ. Eng..

[26] Mansoor S.S., Hama S.M., Hamdullah D.N. (2022). Effectiveness of replacing cement partially with waste brick powder in mortar. J. King Saud Univ..

[27] Darshita T., Anoop P. (2014). Study of Strength and Workability of Different Grades of Concrete by Partial Replacement of Fine Aggregate by Crushed Brick and Recycled Glass Powder. Int. J. Sci. Res..

[28] Aliabdo A.A., Abd-Elmoaty A.E.M., Hassan H.H. (2014). Utilization of crushed clay brick in concrete industry. Alex. Eng. J..

[29] Abdullah D.J., Abbas Z.K., Abed S.K. (2022). Some Properties of Concrete Containing Waste Brick As Partial Replacement Of Coarse Aggregate And Addition Of Nano Brick Powder. IOP Conf. Ser. Earth Environ. Sci..

[30] Liu X., Zhang N. (2011). Utilization of red mud in cement production: A review. Waste Manag. Res..

[31] Akinwekomi A.D., Omotoyinbo J.A., Folorunso D. (2012). Effect of High Alumina Cement on Selected Foundry Properties of Ant-Hill Clay. Leonardo Electron. J. Pract. Technol..

[32] PN M.L., Peter C., Mohan K., Greens S., George S. (2018). Energy efficient production of clay bricks using industrial waste. Heliyon.

[33] Zieliński K., Kierzek D. (2021). The Impact of Alumina Cement on Properties of Portland Cement Slurries and Mortars. Int. J. Struct. Constr. Eng..

[34] Shao J., Gao J., Zhao Y., Chen X. (2019). Study on the pozzolanic reaction of clay brick powder in blended cement pastes. Constr. Build. Mater..

[35] Liu S., Dai R., Cao K., Gao Z. (2017). The Role of Sintered Clay Brick Powder During the Hydration Process of Cement Pastes. Iran. J. Sci. Technol. Trans. Civ. Eng..

[36] Aye T., Oguchi C.T., Takaya Y. (2010). Evaluation of sulfate resistance of Portland and high alumina cement mortars using hardness test. Constr. Build. Mater..

[37] Vejmelková E., Koňáková D., Scheinherrová L., Doleželová M., Keppert M., Černý R. (2018). High temperature durability of fiber reinforced high alumina cement composites. Constr. Build. Mater..

[38] Castillo M., Hernández K., Rodriguez J., Eyzaguirre C. (2019). Low Permeability Concrete for Buildings Located in Marine Atmosphere Zone using Clay Brick Powder. IOP Conf. Ser. Mater. Sci. Eng..

[39] Rani M.U., Jenifer J.M. (2016). Mechanical Properties of Concrete with Partial replacement of Portland Cement by Clay brick powder. Int. J. Eng. Res. Technol..

[40] Zhu P., Mao X., Qu W., Li Z., Ma Z.J. (2016). Investigation of using recycled powder from waste of clay bricks and cement solids in reactive powder concrete. Constr. Build. Mater..

[41] Chaabene W.B., Flah M., Nehdi M.L. (2020). Machine learning prediction of mechanical properties of concrete: Critical review. Constr. Build. Mater..

[42] Czarnecki S., Shariq M., Nikoo M., Sadowski Ł. (2021). An intelligent model for the prediction of the compressive strength of cementitious composites with ground granulated blast furnace slag based on ultrasonic pulse velocity measurements. Measurement.

[43] Czarnecki S., Sadowski Ł., Hoła J. (2020). Artificial neural networks for non-destructive identification of the interlayer bonding between repair overlay and concrete substrate. Adv. Eng. Softw..

[44] Szeląg M. (2021). Intelligent prediction modeling of the post-heating mechanical performance of the brick powder modified cement paste based on the cracking patterns properties. Case Stud. Constr. Mater..

[45] Chen B.T., Chang T.P., Shih J.Y., Wang J.J. (2009). Estimation of exposed temperature for fire-damaged concrete using support vector machine. Comput. Mater. Sci..

[46] (2016). Methods of Testing Cement-Part 1: Determination of Strength.

[47] (2011). Metody badania cementu-Część 6: Oznaczanie stopnia zmielenia.

[48] (2009). Testing Hardened Concrete-Part 5: Flexural Strength of Test Specimens.

[49] (2002). Testing Hardened Concrete-Part 3: Compressive Strength of Test Specimens.

[50] (2001). Metody Badań Zapraw do Murów-Część 10: Określenie Gęstości Wysuszonej Stwardniałej Zaprawy.

[51] Brereton R.G., Lloyd G.R. (2010). Support Vector Machines for classification and regression. Analyst.

[52] He Z.H., Zhu H.N., Zhang M.Y., Shi J.Y., Du S.G., Liu B. (2021). Autogenous shrinkage and nano-mechanical properties of UHPC containing waste brick powder derived from construction and demolition waste. Constr. Build. Mater..

[53] Hay R., Li L., Celik K. (2022). Shrinkage, hydration, and strength development of limestone calcined clay cement (LC3) with different sulfation levels. Cem. Concr. Compos..

[54] Zegardło B., Szeląg M., Ogrodnik P. (2018). Concrete resistant to spalling made with recycled aggregate from sanitary ceramic wastes–The effect of moisture and porosity on destructive processes occurring in fire conditions. Constr. Build. Mater..

[55] Ortega J.M., Letelier V., Solas C., Moriconi G., Climent M.Á., Sánchez I. (2018). Long-term effects of waste brick powder addition in the microstructure and service properties of mortars. Constr. Build. Mater..

[56] Szeląg M., Styczeń J., Fediuk R., Polak R. (2021). Properties and Strength Prediction Modeling of Green Mortar with Brick Powder Subjected to a Short-Term Thermal Shock at Elevated Temperatures. Materials.

[57] Fediuk R., Yushin A. (2016). Modern Technologies of Nondestructive Testing of Construction Materials. IOP Conf. Ser. Mater. Sci. Eng..

